# Supplementary material for: Inflammatory conditions shape phenotypic and functional characteristics of lung-resident memory T cells in mice
Source: Nat Commun. 2025 Apr 16;16:3612. doi: 10.1038/s41467-025-58931-y (PMC12003732; doi:10.1038/s41467-025-58931-y)
Supplement: Supplementary file 1 — Supplementary Information [file 41467_2025_58931_MOESM1_ESM.pdf]

Supplementary Information

Title: Inflammatory conditions shape phenotypic and functional characteristics of lung-resident memory T cells in mice

Authors: Anna Schmidt,…, Matthias Tenbusch

Corresponding: Matthias.Tenbusch@fau.de

Content:

Figures:

Suppl. Fig. 1: Cytokine and chemokine profile in the lung after treatment

Suppl. Fig. 2: Cellular infiltrates in BALF samples

Suppl. Fig. 3: Gating strategy ICS

Suppl. Fig. 4: Gating strategy of CD8+ TRM (pentamer staining)

Suppl. Fig. 5: Functional CD8+ T-cell responses measured by ICS

Suppl. Fig. 6: Gating strategy of CD4+ TRM

Suppl. Fig. 7: Kinetic phenotypic and functional analysis of CD4+ TRM.

Suppl. Fig. 8: Humoral responses against H1N1 A/PR/8/34 and H3N2 A/HK/68

Suppl. Fig. 9: Effect of heterosubtypic H3N2 challenge on functional CD4+ T cells

Suppl. Fig. 10: Bodyweight analysis and viral load after lethal H3N2 challenge in the late memory phase.

Suppl. Fig. 11: Effect of LPS treatment on the fate of pre-existing influenza-specific CD8+ TRM.

Suppl. Fig. 12: *C8a* expression plot, genetic profile of the cells from cluster 3, and cell annotation based on hashtag labeling

Tables:

Suppl. Table 1: Model-based Analysis of Single-cell Transcriptomics (MAST).

Suppl. Table 2: Antibodies used

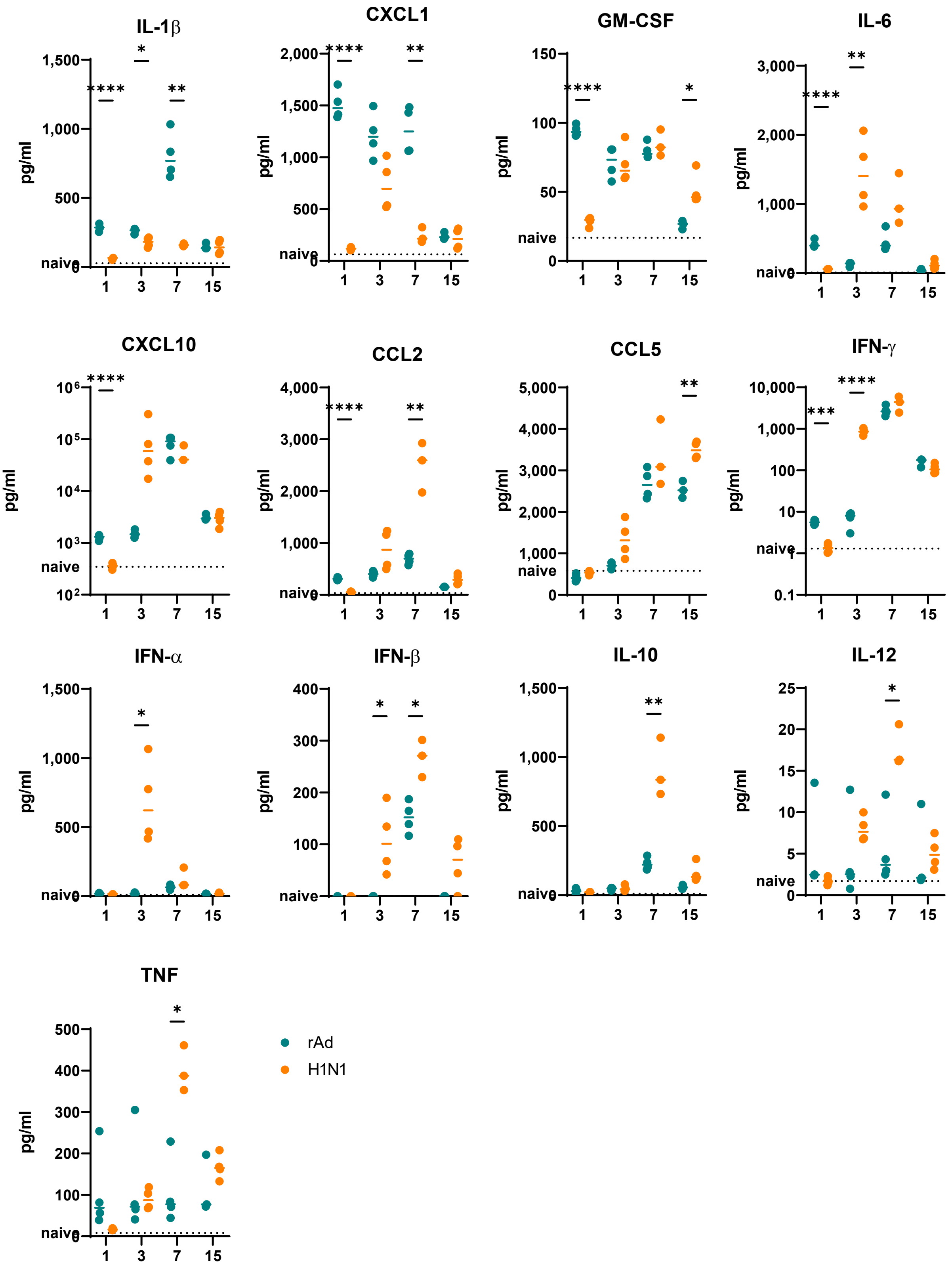

Suppl. Fig. 1: Cytokine and chemokine profile in the lung after treatment. BALB/c mice were either i.n. immunized with rAd-HA, rAd-NP, and rAd-IL-1β (each $2x10^8$ particles) or infected with the H1N1 strain A/PR/8/34 (100 PFU). On days 1,3,7 and 14 post treatment, lungs were removed and homogenized in 1 ml PBS. The levels of thirteen selected cytokines and chemokines were quantified in lung homogenates via a multiplex, cytometric bead assay according to the manufacturers protocol (Legenplex^{TM} mouse anti-virus response panel, BioLegend). Each dots represents an individual animal (n=4 mice per group/time point, except n=3 for rAd (15dpi) and H1N1 (7dpi)). The dotted line indicate the background levels of each analyte in naïve animals (mean of four individual control mice). Statistical significances were analyzed per multiple t-test followed by Holm-Šídák's multiple comparison test (*, p<0.05; **, p<0.01; ***, p<0.001).

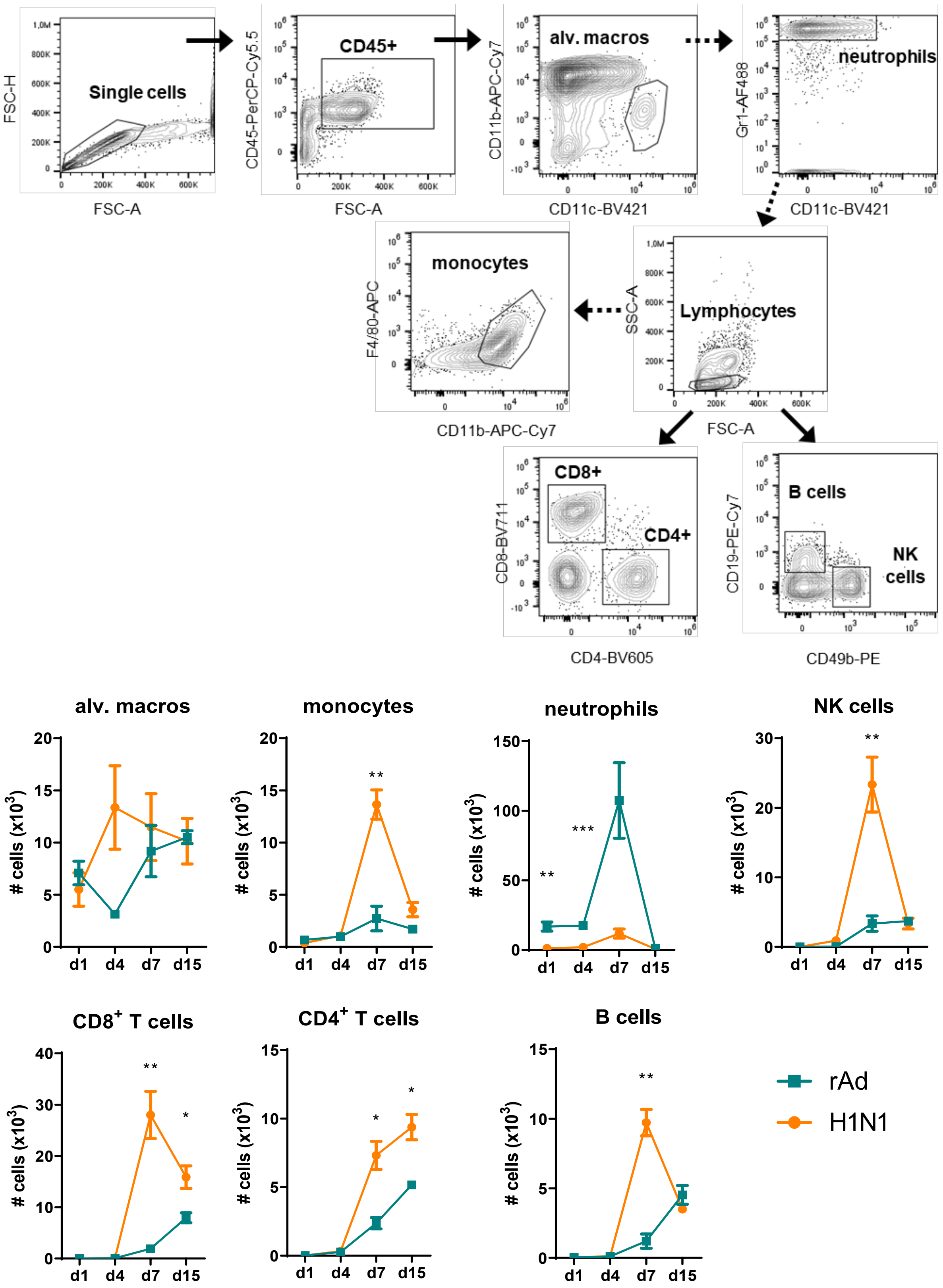

Suppl. Fig. 2: Cellular infiltrates in BALF samples. BALB/c mice were either i.n. immunized with rAd-HA, rAd-NP, and rAd-IL-1β (each $2x10^8$ particles) or infected with the H1N1 strain A/PR/8/34 (100 PFU). On days 1,3,7 and 14 post treatment, BALF were collected and the cellular infiltrate were characterized via flow cytometry according to shown gating. Positive gating is indicated by solid arrows, negative gating (selected population is excluded from next gate) with dashed arrows.

Curves represent the mean with SEM (n=4 mice per group/time point, except n=3 for rAd (1 dpi) and H1N1 (7dpi)). Statistical significances were analyzed per multiple t-test followed by Holm-Šídák's multiple comparison test (*, p<0.05; **, p<0.01; ***, p<0.001).

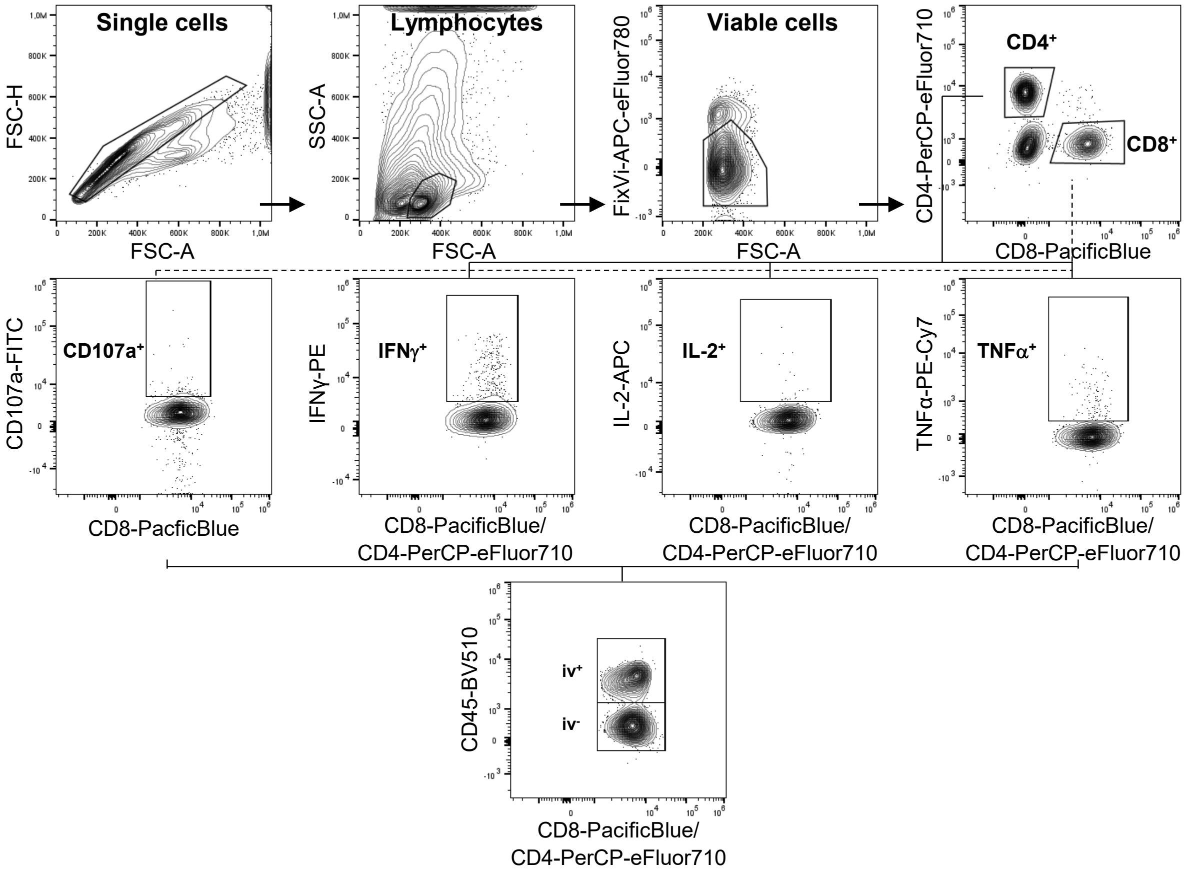

Suppl. Fig. 3: Gating strategy ICS. Single cells and lymphocytes were assorted by virtue of FSC and SSC. Staining of cells with a fixable viability dye (FixVi) allowed the exclusion of dead cells. Subsequently, $CD4^+$ and $CD8^+$ T cells were selected and further analyzed regarding their cytokine production, including IFNγ, IL-2, and TNFα. For $CD8^+$ T cells, degranulation (indicated by CD107a) was additionally evaluated. Systemic and resident proportions were separated by iv-staining of CD45. This gating strategy was used to analyze the cytokine production by antigen-specific T-cells presented in the following graphs of this manuscript: Fig. 2; Fig. 5C+D; Fig. 6D; Suppl. Fig. 5; Suppl. Fig. 7C; Suppl. Fig. 9; Suppl. Fig. 11D.

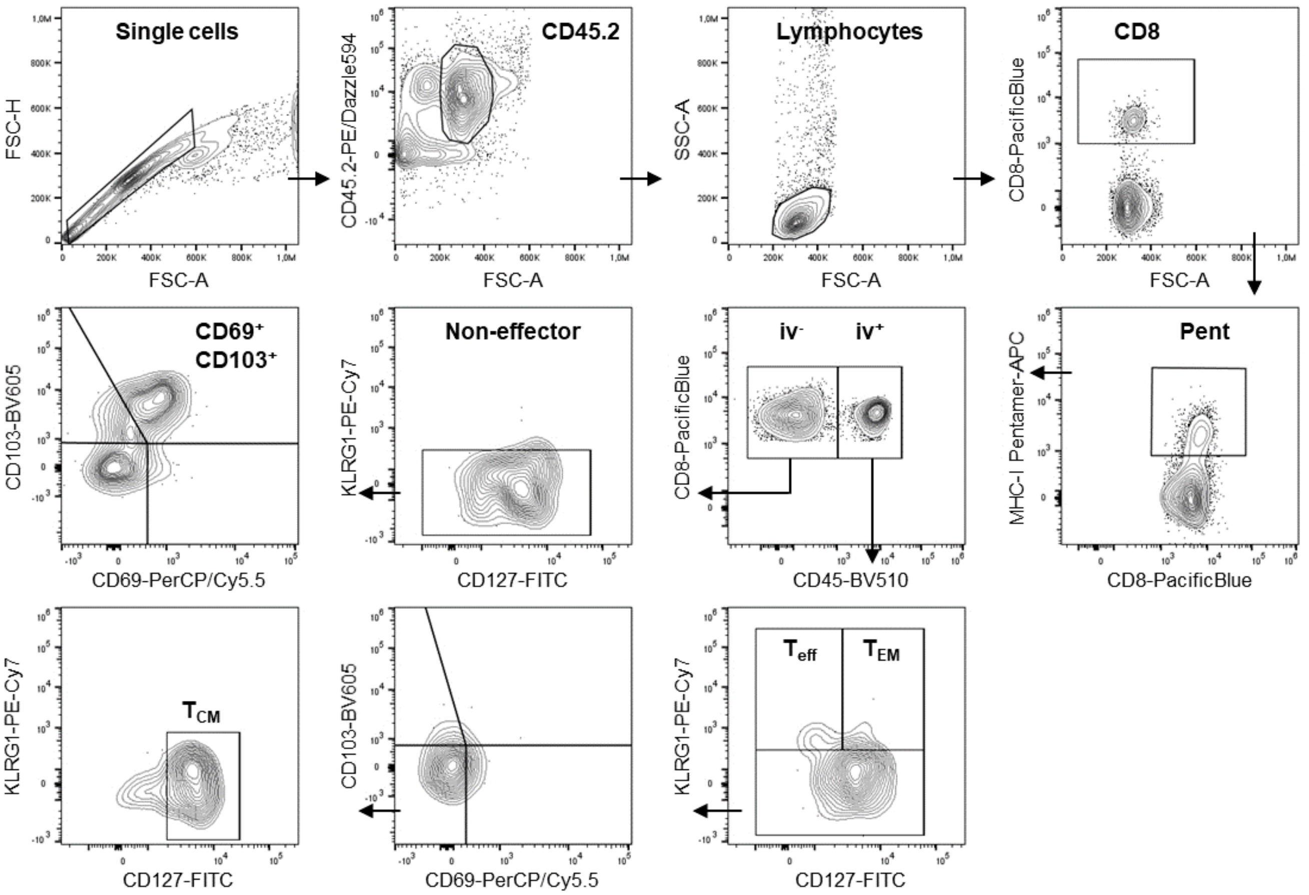

Suppl. Fig. 4: Gating strategy of CD8⁺ T_{RM} (pentamer staining). Single cells were separated, followed by further downscaling of the cell population by gating on CD45.2-positive cells, and through restriction on lymphocytes under application of FSC and SSC. Next, $CD8^+$ T cells were selected and HA- or NP-specific CTLs were identified via MHC-I pentamer staining (Pent). KLRG1 was used in combination with CD127 to separate iv^+ $Pent^+$ $CD8^+$ lymphocytes into effector T cells (T_{eff}, $KLRG1^+$ $CD127^-$), effector memory T cells (T_{EM}, $KLRG1^+$ $CD127^+$), and central memory T cells ($KLRG1^-$ $CD127^+$). The iv^- T_{RM} subtypes were determined using CD69 and CD103. This gating strategy was used to analyze antigen-specific $CD8^+$ T-cells presented in the following graphs of this manuscript: Fig. 1; Fig. 5A+B; Fig. 6C; Suppl. Fig. 11C.

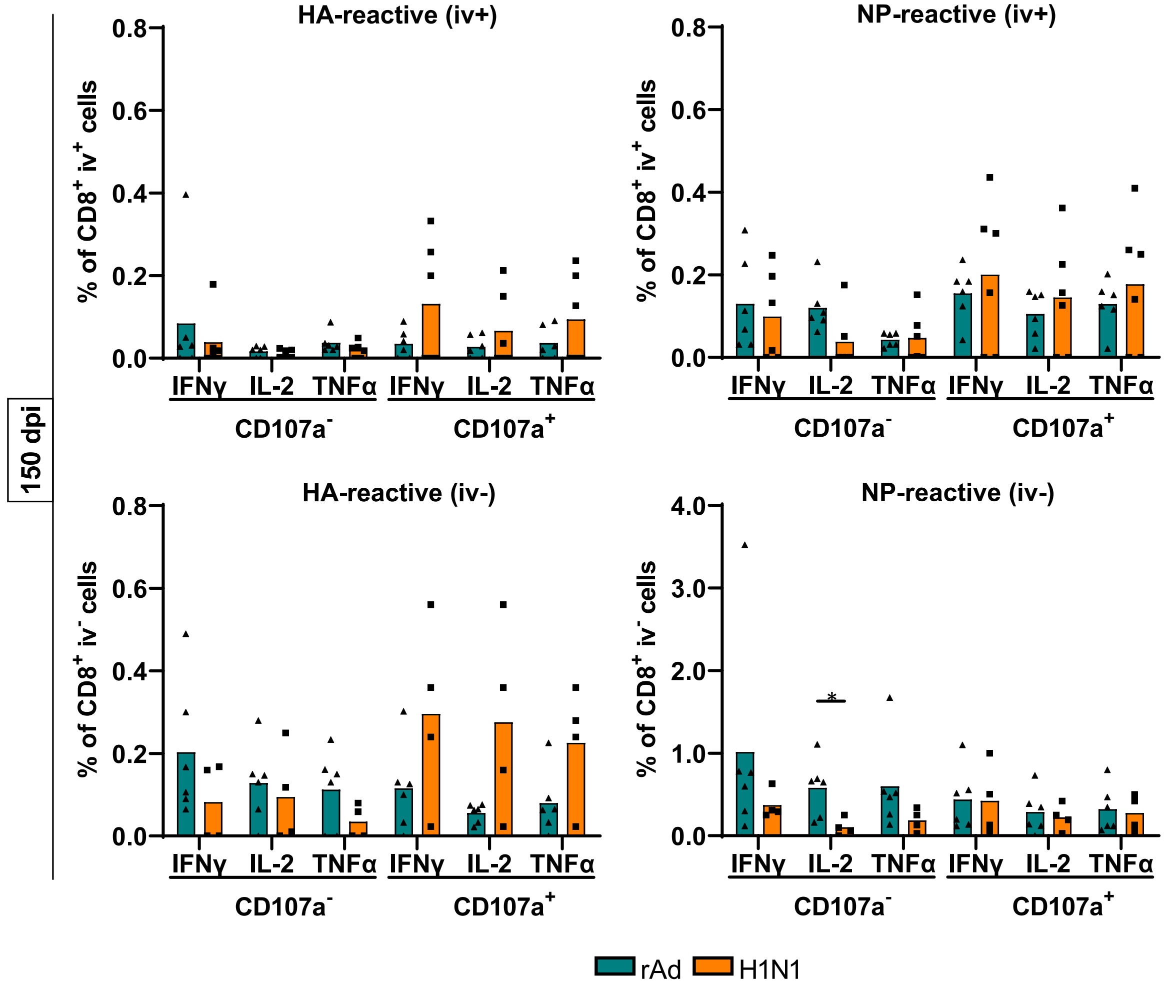

Suppl. Fig. 5: Functional CD8+ T-cell responses measured by ICS. Lymphocytes from rAd-immunized or H1N1-infected mice were isolated at the indicated time points and ICS was used to identify influenza-reactive T cells. Depicted are functional systemic (iv+) and tissue-resident (iv-) CD8+ T-cell subpopulations detected on day 150 after treatment. Frequencies of the respective T-cell populations are shown with each data point representing an individual mouse and bars representing the mean of the group (n=4 mice for H1N1, n=6 mice for rAd). Statistical significances were analyzed by Mann-Whitney test (*, $p<0.05$).

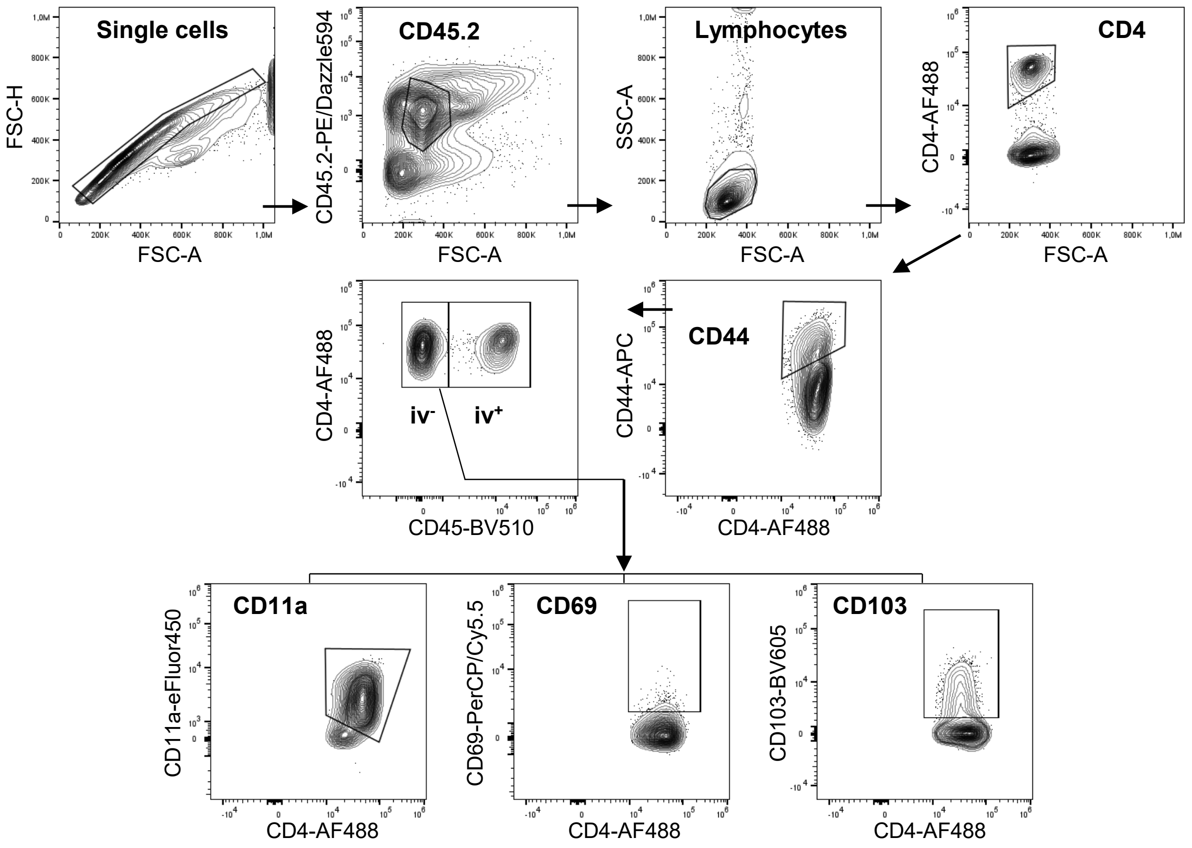

Suppl. Fig. 6: Gating strategy of CD4⁺ T_{RM}. Single cells and CD45.2-positive cells were separated followed by selection of lymphocytes on the basis of FSC and SSC. Subsequently, $CD44^+$ T cells were assorted out of the $CD4^+$ T-cell pool and further subdivided into iv^+ and iv^- cells under application of iv-staining of CD45. Staining for the phenotypic marker CD11a, CD69, and CD103 was used to identify different T_{RM} subpopulations as presented in Suppl. Fig. 7A+B.

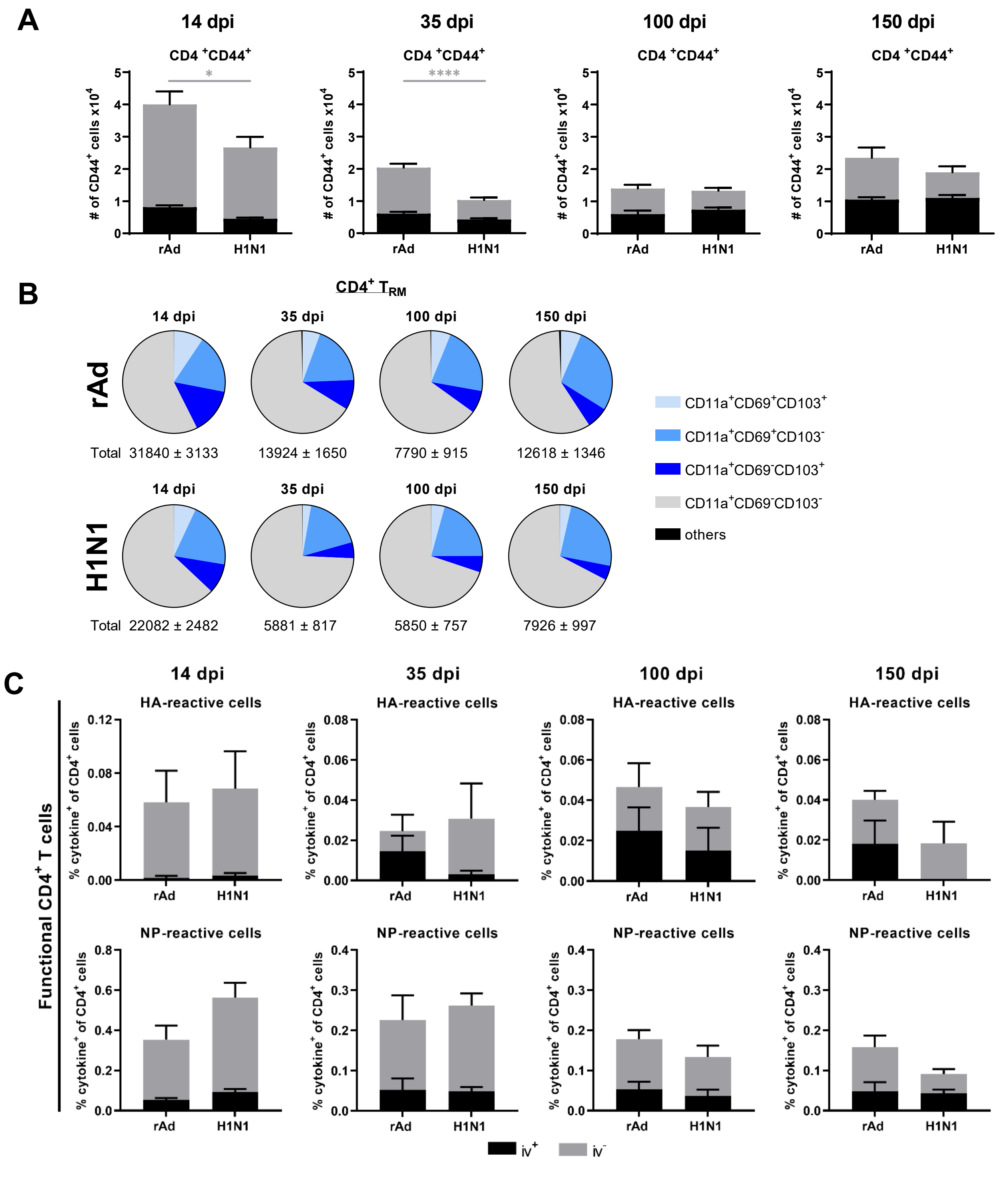

Suppl. Fig. 7: Kinetic phenotypic and functional analysis of CD4⁺ T_{RM}. (A) Depicted are total numbers of activated CD4⁺ T cells identified by staining of CD44, and further separated into iv⁺ and iv⁻ cells. **(B)** The phenotypic occurrence of iv⁻ CD44⁺ lymphocytes was assessed by surface staining of CD11a, CD69, and CD103. The four most common phenotypes observed within the CD4⁺ T-cell population are presented, while iv⁻ memory cells lacking CD11a expression are summarized as others. **(C)** Frequencies of CD4⁺ T cells that are at least positive for the secretion of one specific effector cytokine, including IFNγ, IL-2, and TNFα, are shown for the iv⁺ and the iv⁻ fraction over time. Each data set represents the mean + SEM per group (n=6 mice per group/time point, except 150 dpi in H1N1 group represents only n=4)) . Statistical significances were analyzed by two-way ANOVA followed by Šídák's multiple comparison test (*, p<0.1; ****, p<0.0001 (grey line: iv⁻)).

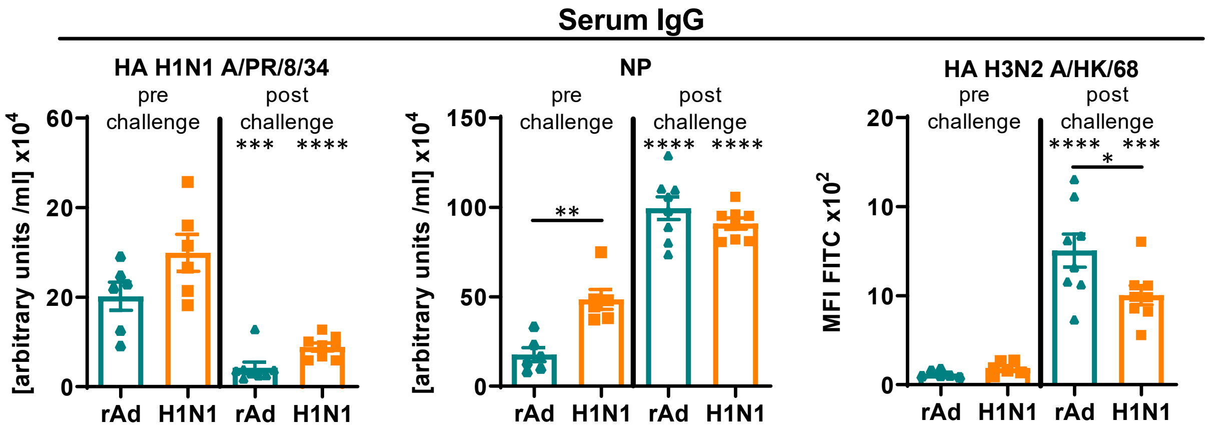

Suppl. Fig. 8: Humoral responses against H1N1 A/PR/8/34 and H3N2 A/HK/68. Antigen-specific IgG antibodies were detected in serum samples (1:200), which were collected on day 55 after the initial prime, and on day 44 after H3N2 challenge. Each data point represents an individual mouse and bars represent the mean of the group (n=6 mice per group). Statistical significances were analyzed by one-way ANOVA followed by Tukey's multiple comparison test and are depicted for pre challenge vs. post challenge or as indicated by lines (*, p<0.1; ***, p<0.001; ****, p<0.0001).

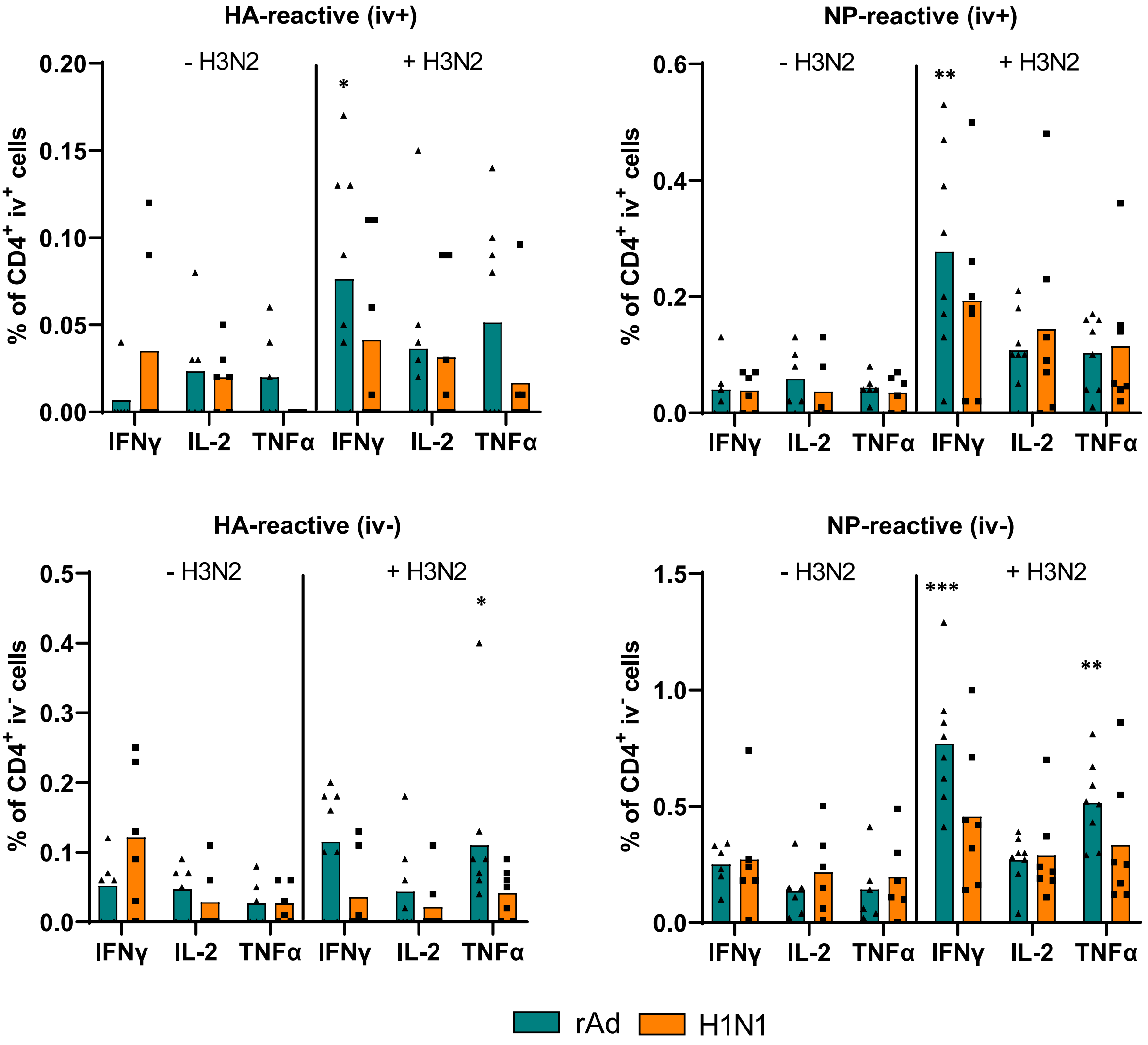

Suppl. Fig. 9: Effect of heterosubtypic H3N2 challenge on functional CD4⁺ T cells. Lymphocytes were isolated from lung tissue of rAd-immunized or H1N1-infected mice on day 100 after primary treatment (-H3N2), and functional T-cell responses were compared with those of rAd-immunized or H1N1-infected mice challenged with H3N2 on day 56 (+ H3N2). Frequencies of cytokine-specific $CD4^{+}$ T cells are shown. Each data point represents an individual mouse and bars represent the mean of the group (n= 6 mice for rAd and H1N1 (-H3N2), 7 mice for H1N1 (+H3N2) and 8 mice for rAd (+ H3N2). To compare statistical effects between unchallenged (- H3N2) and challenged (+ H3N2) mice of one group statistical significances were analyzed by unpaired, two-tailed Mann-Whitney test (*, p<0.05; **, p<0.01; ***, p<0.001).

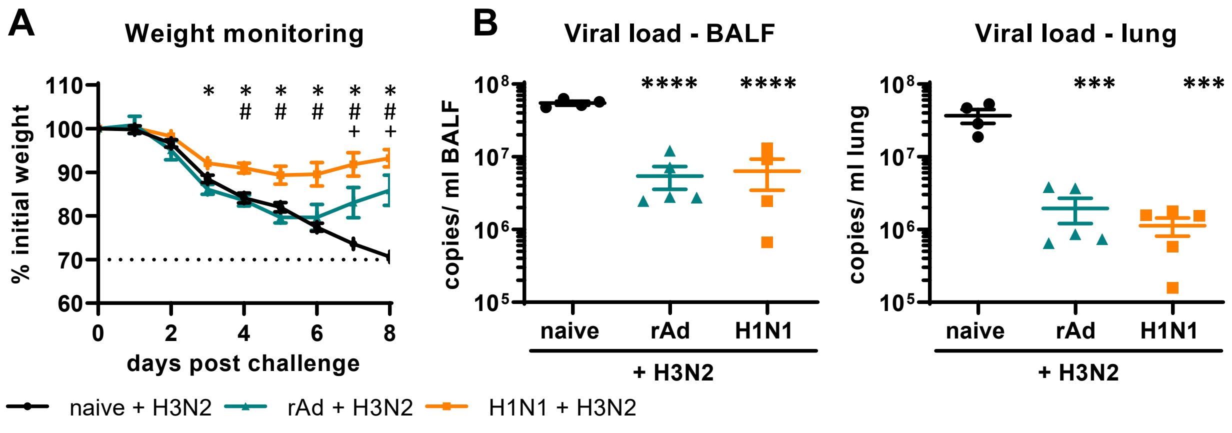

Suppl. Fig. 10: Bodyweight analysis and viral load after lethal H3N2 challenge in the late memory phase. Previously rAd-immunized or H1N1-infected mice were challenged with the heterosubtypic H3N2 on day 216 after priming and **(A)** bodyweight was monitored daily. Depicted are percentages of the initial weight on day 0. **(B)** Viral load in BALF and lung samples from day eight after H3N2 challenge was assessed by qRT-PCR. **(A, B)** Each data point represents an individual mouse and the mean + SEM are indicated by lines and error bars (n=4 mice for naïve, n= 5 for rAd and H1N1). Statistical significances were analyzed by (A) two-way ANOVA followed by Tukey's multiple comparison test or by (B) one-way ANOVA followed by Tukey's multiple comparison test ((A) *, p<0.05 H1N1 vs. rAd; #, p<0.05 H1N1 vs. naïve; +, p<0.05 rad vs. naïve; (B): ***, p<0.001 vs. naïve; ****, p<0.0001 vs. naïve).

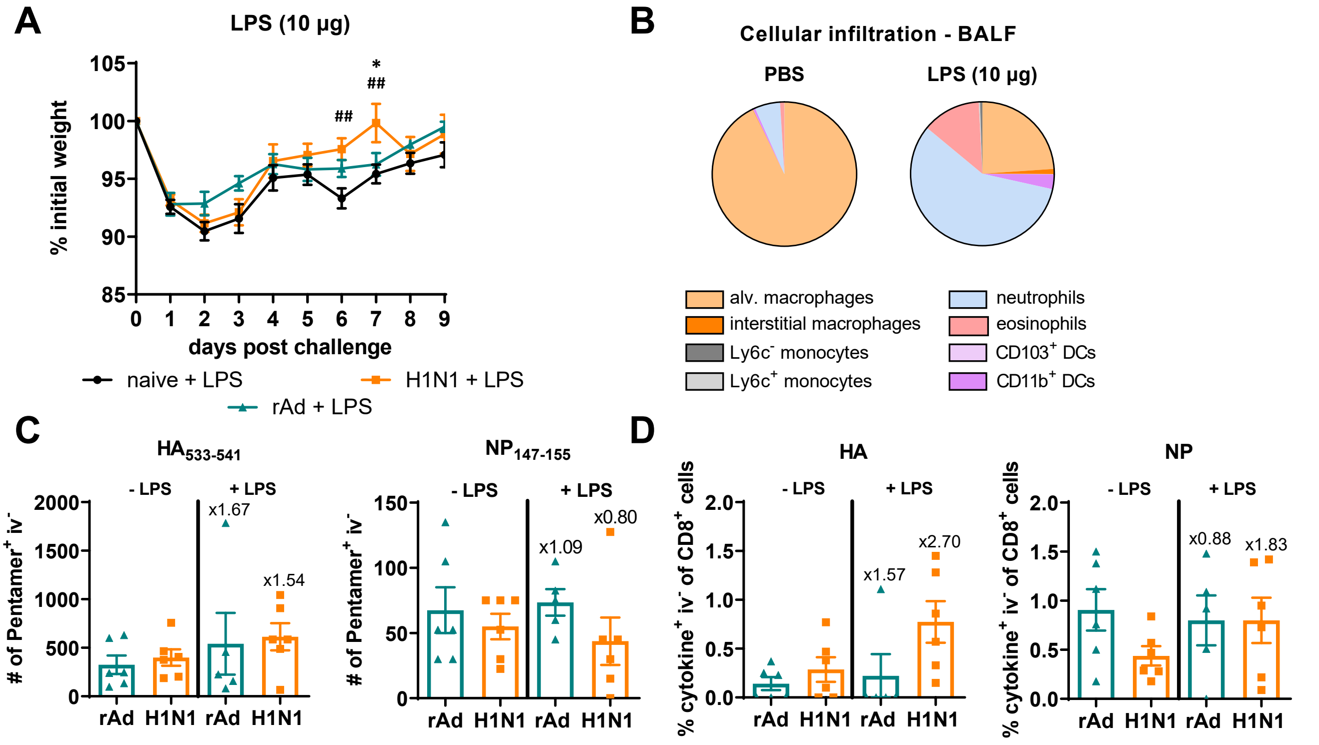

Suppl. Fig. 11: Effect of LPS treatment on the fate of pre-existing influenza-specific CD8⁺ T_{RM}.
Mice were rAd-immunized or H1N1-infected as described before and inoculated with 10 µg LPS i.n. on day 56 after priming (+ LPS). One set of mice remained unchallenged and served as control group (- LPS). **(A)** Body weight was measured daily for nine days after challenge. **(B)** One extra set of mice was challenged with 10 µg LPS on day 68 after immunization and the immune cell infiltration in BALF was measured after 72 h. PBS was given as control. Data represent the mean of three mice per treatment (n=3). **(C)** Three weeks after LPS administration, lymphocytes from lung tissues were isolated to determine total numbers of $HA_{533\text{-}541}$- and $NP_{147\text{-}155}$-specific tissue-resident $CD8^+$ T cells. **(D)** Part of the lymphocytes were restimulated *in vitro* and frequencies of iv^- $CD8^+$ T cells expressing either CD107a, IFNγ, IL-2, and/or TNFα are shown. **(A)** Values are shown as percentage of the initial weight on day 0 or **(C, D)** are represented as total numbers of $Pent^+$ $CD8^+$ T cells or frequencies of cytokine producing iv^- $CD8^+$ T cells. Each data point represents an individual mouse and the mean + SEM are indicated by lines and error bars (n=6 mice per group, except n= 5 for rAd (+LPS)). Statistical significances were analyzed **(A)** by two-way ANOVA followed by Tukey's multiple comparison test or **(C, D)** by one-way ANOVA followed by Tukey's multiple comparison test (*, p<0.1 H1N1+LPS vs. rAd+LPS, ##, p<0.01 H1N1+LPS vs. naïve+LPS).

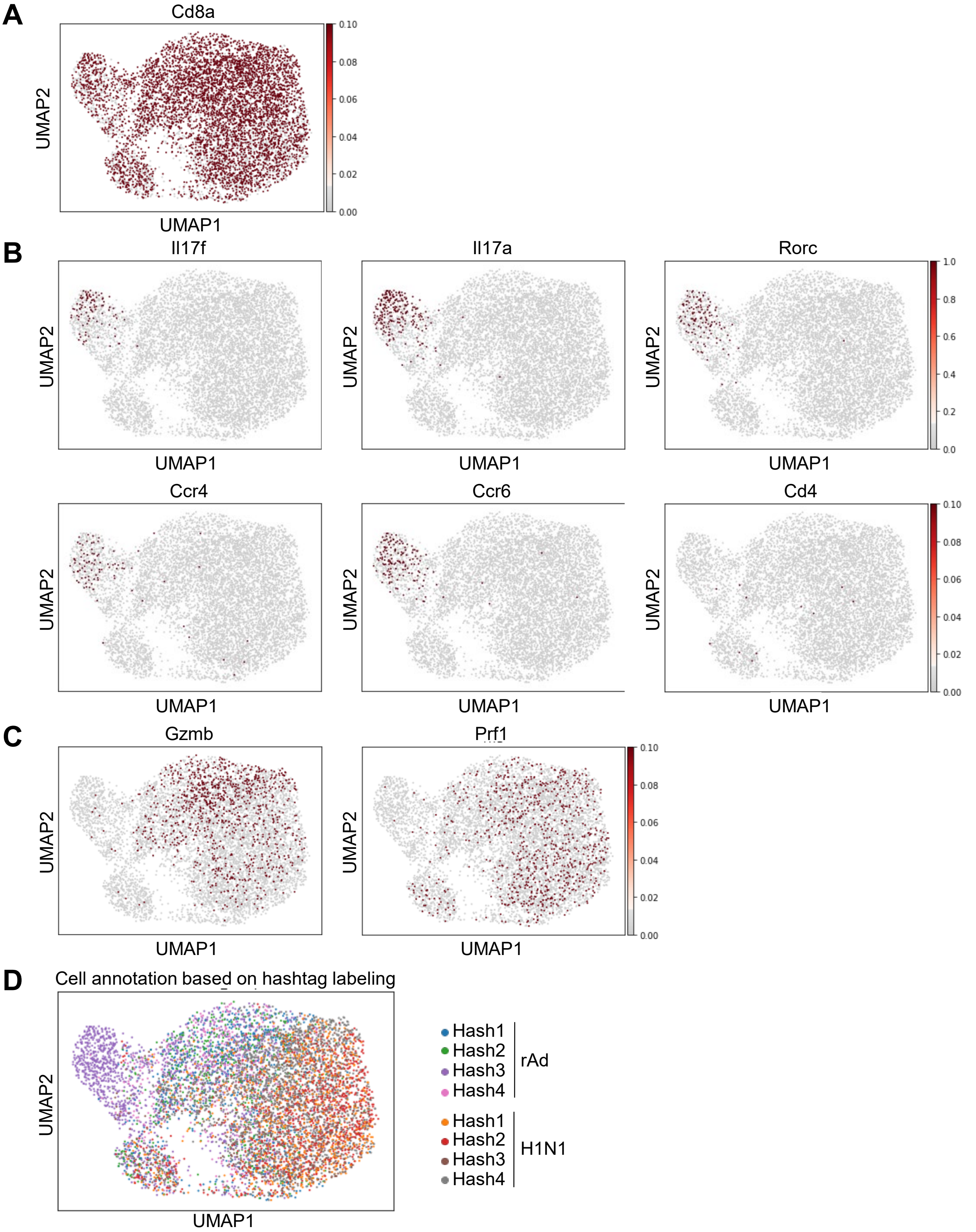

Suppl. Fig. 12: *Cd8a* expression plot, genetic profile of the cells from cluster 3, and cell annotation based on hashtag labeling. (A, B, C) UMAP plots of *Cd8a* and further indicated genes including all cells regardless of the treatment group. The expression of the indicated genes is represented by the normalized UMI count. The maximum displayed normalized UMI count for the UMAP plots has been set to 0.1 or 1, respectively, and all cells exceeding an expression of these values are downsized to the respective value for visualization purposes. **(D)** Assignment of each cell barcode to one corresponding mouse. Hash 1-4 is related to four animals of the different treatment groups. Colored circles reflect the cluster assignment identified by Leiden.

Suppl. Table 1: Model-based Analysis of Single-cell Transcriptomics (MAST).

**Immunized mice**	**Differentially expressed genes (from a total of 15754 genes)**	
rAd_3 vs. rAd_1	301 = 1.91 %	Average 1.34 %
rAd_3 vs. rAd_2	188 = 1.14 %	
rAd_3 vs. rAd_4	153 = 0.97%	
rAd_4 vs. rAd_1	41 = 0.26 %	Average 0.10 %
rAd_4 vs. rAd_2	30 = 0.19 %	
rAd_1 vs. rAd_2	24 = 0.15 %	
**Infected mice**		
H1N1_2 vs. H1N1_4	532 = 3.38 %	
H1N1_2 vs. H1N1_1	223 = 1.42 %	
H1N1_2 vs. H1N1_3	188 = 1.19 %	
H1N1_4 vs. H1N1_1	176 = 1.12 %	
H1N1_4 vs. H1N1_3	131 = 0.83 %	
H1N1_1 vs. H1N1_3	28 = 0.18 %	

Suppl. Table 2: Antibodies used.

Antigen	Clone	Isotype	Fluorochrome	Source (Cat.)	Dilution
**Intracellular cytokine staining**					
CD3ε	145-2C11	Armenian Hamster IgG1, κ	-	BD Biosciences (Cat: 553057)	2 µg/ml
CD4	RM4-5	Rat IgG2a, κ	PerCP-eFluor710	invitrogen (Cat: 46-0042-82)	1:2000
CD8α	53-6.7	Rat IgG2a, κ	Pacific Blue	BioLegend (Cat: 100725)	1:300
CD16/ CD32	93	Rat IgG2a, λ	-	invitrogen (Cat: 14-0161-86)	2 µg/ml
CD28	37.51	Syrian Hamster IgG	-	invitrogen (Cat: 14-0282-86)	1 µg/ml
CD45	30-F11	Rat IgG2b, κ	BV510	BioLegend (Cat: 103138)	2 µg
CD107a	1D4B	Rat IgG2a, κ	FITC	BD Biosciences (Cat: 553793)	1:100
IFNγ	XMG1.2	Rat IgG1, κ	PE	BioLegend (Cat: 505808)	1:300
IL-2	JES6-5H4	Rat IgG2b, κ	APC	BioLegend (Cat: 503810)	1:300
TNFα	MP6-XT22	Rat IgG1, κ	PE-Cy7	BioLegend (Cat: 506324)	1:300
**Pentamer staining**					
CD4	GK1.5	Rat IgG2b, κ	AF488	BioLegend (Cat: 100423)	1:200
CD8α	53-6.7	Rat IgG2a, κ	Pacific Blue	BioLegend (Cat: 100725)	1:300
CD11a	M17/4	Rat IgG2a, κ	eFluor450	invitrogen (Cat: 48-0111-82)	1:300
CD44	IM7	Rat IgG2b, κ	APC	BioLegend (Cat: 103018)	1:200
CD45	30-F11	Rat IgG2b, κ	BV510	BioLegend (Cat: 103138)	2 µg
CD45.2	104	Mouse (SJL)IgG2a, κ	PE/Dazzle594	BioLegend (Cat: 109846)	1:500
CD69	H1.2F3	Armenian Hamster IgG	PerCP/Cy5.5	BioLegend (Cat: 104522)	1:200
CD103	2E7	Armenian Hamster IgG	BV605	BioLegend (Cat: 121433)	1:200
CD127 (Il-7Ra)	A7R34	Rat IgG2a, κ	FITC	BioLegend (Cat: 135008)	1:300
CXCR3	173	Armenian Hamster IgG	APC-Fire750	BioLegend (Cat: 126539)	1:200
IFITM3	aa2-57	Goat IgG	Biotin	R&D Systems (Cat: BAF3377)	1:300
KLRG1	2F1	Syrian Hamster IgG	PE-Cy7	invitrogen (Cat: 25-5893-82)	1:300
P2XR7	1F11	Rat IgG2b, κ	PE	BioLegend (Cat: 148704)	1:300
Streptavidin			BV711	BioLegend (Cat: 405241)	1:300

Suppl. Table 2: Antibodies used (continued).

**Antibody binding assay staining**					
IgA	polyclonal	Goat IgG	FITC	Fortis Life Sciences (Cat: A90-103F)	1:300
IgG	Poly4060	Rat Polyclonal IgG	FITC	BioLegend (Cat: 406001)	1:300
IgG1	RMG1-1	Rat IgG	APC	BioLegend (Cat: 406610)	1:300
IgG2a	m2a-15F8	Rat IgG1, κ	PerCP-eFluor710	Invitrogen (Cat: 46-4210-82)	1:300
**Cell sorting staining**					
CD8α	53-6.7	Rat IgG2a, κ	BV421	BioLegend (Cat: 100737)	1:300
CD45.2	104	Mouse (SJL)IgG2a, κ	PE/Dazzle594	BioLegend (Cat: 109846)	1:300
**Immunofluorescence staining**					
CD4	GK1.5	Rat IgG2b, κ	AF488	BioLegend (Cat: 100423)	5 µg/ml
CD8α	53-6.7	Rat IgG2a, κ	AF488	BD Biosciences (Cat: 557668)	5 µg/ml
CD8α	53-6.7	Rat IgG2a, κ	AF647	BioLegend (Cat: 100727)	5 µg/ml
CD16/ CD32	93	Rat IgG2a, λ	-	invitrogen (Cat: 14-0161-86)	10 µg/ml
CD45R/B220	RA3-6B2	Rat IgG2a, κ	AF488	BioLegend (Cat: 103225)	5 µg/ml
CD45R/B220	RA3-6B2	Rat IgG2a, κ	BV711	BioLegend (Cat: 103255)	5 µg/ml
Allophycocyanin	936809	Mouse IgG2b	AF647	R&D Systems (Cat: FAB8927R)	10 µg/ml
Mouse IgG2b	polyclonal	Goat IgG	AF647	invitrogen (Cat: A21242)	30 µg/ml
Streptavidin			PE	Miltenyi (Cat: 130-106-790)	1:2000
**Infiltrating immune cell staining (BALF)**					
CD11c	HL3	Armenian Hamster IgG1, λ2	BV421	BD Biosciences, (Cat: 560521)	1:200
Gr-1	RB6-8C5	Rat IgG2b, κ	AF488	BioLegend (Cat: 108417)	1:300
CD49b	DX5	Rat / IgM, kappa	PE	BioLegend (Cat: 108907)	1:300
CD45	30-F11	Rat IgG2b, κ	PerCP-Cy5.5	BD Biosciences (Cat: 550994)	1:300
CD19	1D3	Rat IgG2a, κ	PE-Cy7	BD Biosciences (Cat: 552854)	1:1000
F4/80	BM8	Rat IgG2a, κ	APC	BioLegend (Cat: 123116)	1:300
CD11b	M1/70	Rat IgG2b, κ	APC-Cy7	BD Biosciences (Cat: 557657)	1:300
CD4	RM4-5	Rat IgG2a, κ	BV605	BioLegend (Cat: 100547)	1:300
CD8	53-6.7	Rat IgG2a, κ	BV711	BioLegend (Cat: 100747)	1:300
CD3ε	145-2C11	Armenian Hamster IgG	BV510	BioLegend (Cat: 100353)	1:200
