# Supplementary material for: Inflammatory conditions shape phenotypic and functional characteristics of lung-resident memory T cells in mice
Source: Nat Commun. 2025 Apr 16;16:3612. doi: 10.1038/s41467-025-58931-y (PMC12003732; doi:10.1038/s41467-025-58931-y)
Supplement: Supplementary file 2 — Description of Additional Supplementary Files [file 41467_2025_58931_MOESM2_ESM.pdf]

Description of Additional Supplementary Files

Supplementary Data 1: Significant differentially expressed genes in the TRM compartment between vaccinated and infected mice. Genes are sorted by -log10(value). (A) A negative fold change (FC) value indicates a decreased expression in infected mice compared to vaccinated ones and thus an enhanced expression of the respective gene in mice of the rAd group. (B) A positive FC indicates an increased expression in infected mice.

Supplementary Data 2: Top ten TRM clonotype sequences per group identified via Scirpy.

Supplementary Data 3: Clonotype sequences shared between rAd and H1N1 identified via Scirpy.
