# Supplementary material for: Inflammatory conditions shape phenotypic and functional characteristics of lung-resident memory T cells in mice
Source: Nat Commun. 2025 Apr 16;16:3612. doi: 10.1038/s41467-025-58931-y (PMC12003732; doi:10.1038/s41467-025-58931-y)
Supplement: Supplementary file 6 — Reporting Summary [file 41467_2025_58931_MOESM6_ESM.pdf]

nature portfolio

Corresponding author(s): Matthias Tenbusch

Last updated by author(s): Feb 21, 2025

Reporting Summary

Nature Portfolio wishes to improve the reproducibility of the work that we publish. This form provides structure for consistency and transparency in reporting. For further information on Nature Portfolio policies, see our Editorial Policies and the Editorial Policy Checklist.

Statistics

For all statistical analyses, confirm that the following items are present in the figure legend, table legend, main text, or Methods section.

n/a	Confirmed	
[ ]	[x]	The exact sample size (*n*) for each experimental group/condition, given as a discrete number and unit of measurement
[ ]	[x]	A statement on whether measurements were taken from distinct samples or whether the same sample was measured repeatedly
[ ]	[x]	The statistical test(s) used AND whether they are one- or two-sided<br>*Only common tests should be described solely by name; describe more complex techniques in the Methods section.*
[x]	[ ]	A description of all covariates tested
[ ]	[x]	A description of any assumptions or corrections, such as tests of normality and adjustment for multiple comparisons
[ ]	[x]	A full description of the statistical parameters including central tendency (e.g. means) or other basic estimates (e.g. regression coefficient) AND variation (e.g. standard deviation) or associated estimates of uncertainty (e.g. confidence intervals)
[ ]	[x]	For null hypothesis testing, the test statistic (e.g. *F*, *t*, *r*) with confidence intervals, effect sizes, degrees of freedom and *P* value noted<br>*Give P values as exact values whenever suitable.*
[x]	[ ]	For Bayesian analysis, information on the choice of priors and Markov chain Monte Carlo settings
[x]	[ ]	For hierarchical and complex designs, identification of the appropriate level for tests and full reporting of outcomes
[x]	[ ]	Estimates of effect sizes (e.g. Cohen's *d*, Pearson's *r*), indicating how they were calculated

Our web collection on statistics for biologists contains articles on many of the points above.

Software and code

Policy information about availability of computer code

Data collection	Applied Biosystems 7500 Real Time PCR System<br>Beckmann Coulter MoFlo Astrios Cell Sorter<br>Hamamatsu S210 digital slide scanner<br>Leica Confocal microscope, SP5X laser scanning<br>Starr Life Science MouseOx Pulse-oximeter<br>ThermoFisher AttuneNxt v3.2.1526.0

Data analysis	Cell Ranger v.6.1.2<br>DE Seq2 v.1.24.0<br>FlowJo v.10.8.1<br>FlowLogic v.7.2.1<br>GraphPad Prism v.9.5.1<br>ImageJ v.1.47<br>LAS AF Lite v.2.6.0 build 7266<br>Loupe Browser v.4.0<br>QuPath v.0.4.2<br>Scanpy v.1.9.3<br>Scirpy v.0.11.2<br>scSELpy v.1.1.9<br>scType<br>7500 software v.2.3

For manuscripts utilizing custom algorithms or software that are central to the research but not yet described in published literature, software must be made available to editors and reviewers. We strongly encourage code deposition in a community repository (e.g. GitHub). See the Nature Portfolio guidelines for submitting code & software for further information.

Data

Policy information about availability of data

All manuscripts must include a data availability statement. This statement should provide the following information, where applicable:

- Accession codes, unique identifiers, or web links for publicly available datasets
- A description of any restrictions on data availability
- For clinical datasets or third party data, please ensure that the statement adheres to our policy

The sequencing dataset have been deposited at Gene Expression Omnibus (GEO) under the series accession number GSE261708. All data are included in the Supplementary Information or available from the authors, as are unique reagents used in this Article. The raw numbers for charts and graphs are available in the Source Data file whenever possible.

Field-specific reporting

Please select the one below that is the best fit for your research. If you are not sure, read the appropriate sections before making your selection.

☒ Life sciences ☐ Behavioural & social sciences ☐ Ecological, evolutionary & environmental sciences

For a reference copy of the document with all sections, see nature.com/documents/nr-reporting-summary-flat.pdf

Life sciences study design

All studies must disclose on these points even when the disclosure is negative.

Sample size	Sample size was determined by power analysis based on existing data sets from previous experiments with similar readouts and expected statistical errors (type I and II errors 0.05). Where no appropriate data sets existed, the minimal number of animals was choosen in order to produce data with sufficient statistical power.
Data exclusions	No data was excluded.
Replication	4-8 mice were included for each control, vaccination or infection group. Immune responses, viral loads, and monitoring parameters upon infection were tested in all individual group animals. Some tests were repeated several times. All attempts at replication were successful.
Randomization	Mice were randomly allocated to experimental groups by the animal care taker upon delivery.
Blinding	Blinding was not performed due to limited number of staff available to conduct these studies. Where possible, staff was blinded to groupings until after generation of the raw data.

Reporting for specific materials, systems and methods

We require information from authors about some types of materials, experimental systems and methods used in many studies. Here, indicate whether each material, system or method listed is relevant to your study. If you are not sure if a list item applies to your research, read the appropriate section before selecting a response.

Materials & experimental systems

n/a	Involved in the study
	☒ Antibodies
	☒ Eukaryotic cell lines
☒	Palaeontology and archaeology
	☒ Animals and other organisms
☒	Human research participants
☒	Clinical data
☒	Dual use research of concern

Methods

n/a	Involved in the study
☒	ChIP-seq
	☒ Flow cytometry
☒	MRI-based neuroimaging

Antibodies

Antibodies used

anti-CD3e-BV510, clone 145-2C11, BioLegend, Cat: 100353, Lot: B374413
anti-CD4-BV605, clone RM4-5, BioLegend, Cat: 100547, Lot: B380546
anti-CD8-BV711, clone 53-6.7, BioLegend, Cat: 100747, Lot: B415531
anti-CD11b-APC-Cy7, clone M1/70, BD Biosciences, Cat: 557657, Lot: 7278813
anti-CD11c-BV421, clone HL3, BD Biosciences, Cat: 560521, Lot: 4053297
anti-CD19-PE-Cy7, clone 1D3, BD Biosciences, Cat: 552854, Lot: 7194589
anti-CD45-PerCP-Cy5.5, clone 30-F11, BD Biosciences, Cat: 550994, Lot: 9249193
anti-CD49b-PE, clone DX5, BioLegend, Cat: 108907, Lot: B278345
anti-F4/80-APC, clone BM8, BioLegend, Cat: 123116, Lot: B321485
anti-Gr-1.AF488, clone RB6-8C5, BioLegend, Cat: 108417, Lot: B297805

anti-CD3e, clone 145-2C11, BD Biosciences, Cat: 553057, Lot: 0121757
anti-CD4-PerCP-eFluor710, clone RM4-5, invitrogen, Cat: 46-0042-82, Lot: 4317308
anti-CD8a-Pacific Blue, clone 53-6.7, BioLegend, Cat: 100725, Lot: B340544
anti-CD16/CD32, clone 93, invitrogen, Cat: 14-0161-86, Lot: 2297433
anti-CD28, clone: 37.51, invitrogen, Cat: 14-0281-86, Lot: 2124568
anti-CD45-BV510, clone 30-F11, Biolegend, Cat: 103138, Lot: B386738
anti-CD107a-FITC, clone eBio1D4B, BD Bioscience, Cat: 553793, Lot: 1356288
anti-IFNγ-PE, clone XMG1.2, BioLegend, Cat: 505808, Lot: B352685
anti-IL-2-APC, clone JES6-5H4, BioLegend, Cat: 503810, Lot: B281632
anti-TNFα-PE-Cy7, clone MP6-XT22, BioLegend, Cat: 506324, Lot: B338090

anti-CD4-AF488, clone GK1.5, BioLegend, Cat: 100423, Lot: B256618
anti-CD11a-eFluor450, clone M17/4, invitrogen, Cat: 48-0111-82, Lot: 1935030
anti-CD44-APC, clone IM7, BioLegend, Cat: 103018, Lot: B317762
anti-CD45.2-PE/Dazzle594, clone 104, BioLegend, Cat: 109846, Lot: B319002
anti-CD69-PerCP/Cy5.5, clone H1.2F3, BioLegend, Cat: 104522, Lot: B287423
anti-CD103-BV605, clone 2E7, BioLegend, Cat: 121433, Lot: B330662
anti-CD127-FITC, clone A7R34, BioLegend, Cat: 135008, Lot: B255263
anti-CXCR3-APC-Fire750, clone 173, BioLegend, Cat: 126539, Lot: B319572
anti-IFITM3-Biotin, clone aa2-57, R&D Systems, Cat: BAF3377, Lot: XHC016041
anti-KLRG1-PE-Cy7, clone 2F1, invitrogen, Cat: 25-5893-82, Lot: 1982690
anti-P2X7R-PE, clone 1F11, BioLegend, Cat: 148704, Lot: B278527
Streptavidin-BV711, BioLegend, Cat: 405241, Lot: B370754

anti-mouse IgA-FITC, Fortis Life Sciences, Cat: A90-103F, Lot: 21
anti-mouse IgG-FITC, clone Poly4060, BioLegend, Cat: 406001, Lot: B325073
anti-mouse IgG1-APC, clone RMG1-1, BioLegend, Cat:406610, Lot: B302145
anti-mouse IgG2a-PerCP-eFluor710, clone m2a-15F8, invitrogen, Cat: 46-4210-82, Lot: 2464459

TotalSeqTM-C0301 anti-mouse Hashtag 1 Antibody, BioLegend, Cat: 155861, Lot: B362113
TotalSeqTM-C0302 anti-mouse Hashtag 2 Antibody, BioLegend, Cat: 155863, Lot: B375383
TotalSeqTM-C0303 anti-mouse Hashtag 3 Antibody, BioLegend, Cat: 155865, Lot: B368268
TotalSeqTM-C0304 anti-mouse Hashtag 4 Antibody, BioLegend, Cat: 155867, Lot: B361361
anti-CD8a-BV421, clone 53-6.7, BioLegend, Cat: 100737, Lot: B284315

anti-CD8a-AF488, clone 53-6.7, BD Biosciences, Cat: 557668, Lot: 2070069
anti-CD8a-AF674, clone 53-6.7, BioLegend, Cat: 100727, Lot: B375103
anti-CD45R/B220-AF488, clone RA3-6B2, BioLegend, Cat: 103225, Lot: B235299
anti-CD45R/B220-BV711, clone RA3-6B2, BioLegend, Cat: 103255, Lot: B326247
anti-APC-AF647, clone 936809, R&D Systems, Cat: FAB8927R, Lot: AFJR0120061
anti-mouseIgG2b-AF647, invitrogen, Cat: A21242, Lot: 2465093

Streptavidin-PE, Miltenyi, Cat: 130-106-790, Lot: 5230309005

Validation

All antibodies are commercially available and were validated by the manufacturer as follows:

anti-CD3e-BV510, clone 145-2C11, BioLegend, Cat: 100353, Lot: B374413
The manufacturer provides the following statement: Each lot of this antibody is quality control tested by immunofluorescent staining with flow cytometric analysis. For flow cytometric staining, the suggested use of this reagent is ≤0.5 µg per million cells in 100 µl volume. It is recommended that the reagent be titrated for optimal performance for each application. The manufacturer states that the antibody was used in 12 publications.

anti-CD4-BV605, clone RM4-5, BioLegend, Cat: 100547, Lot: B380546
The manufacturer provides the following statement: Each lot of this antibody is quality control tested by immunofluorescent staining with flow cytometric analysis. For immunofluorescent staining using the µg size, the suggested use of this reagent is ≤0.25 µg per million cells in 100 µl volume. For immunofluorescent staining using the µl size, the suggested use of this reagent is 5 µl per million cells in 100 µl staining volume or 5 µl per 100 µl of whole blood. It is recommended that the reagent be titrated for optimal performance for each application. The manufacturer states that the antibody was used in 116 publications.

anti-CD8-BV711, clone 53-6.7, BioLegend, Cat: 100747, Lot: B415531
The manufacturer provides the following statement: Each lot of this antibody is quality control tested by immunofluorescent staining with flow cytometric analysis. For immunofluorescent staining using the µg size, the suggested use of this reagent is ≤0.5 µg per million cells in 100 µl volume. For immunofluorescent staining using µl sizes, the suggested use of this reagent is 5 µl per million cells in 100 µl staining volume or 5 µl per 100 µl of whole blood. It is recommended that the reagent be titrated for optimal performance for each application. The manufacturer states that the antibody was used in 85 publications.

anti-CD11b-APC-Cy7, clone M1/70, BD Biosciences, Cat: 557657, Lot: 7278813
The manufacturer provides the following statement: Flow cytometry (Routinely Tested). The manufacturer states that the antibody was used in 239 publications.

anti-CD11c-BV421, clone HL3, BD Biosciences, Cat: 560521, Lot: 4053297
The manufacturer provides the following statement: Flow cytometry (Routinely Tested), Immunofluorescence (Tested During Development). The manufacturer states that the antibody was used in 38 publications.

anti-CD19-PE-Cy7, clone 1D3, BD Biosciences, Cat: 552854, Lot: 7194589
The manufacturer provides the following statement: Flow cytometry (Routinely Tested). The manufacturer states that the antibody was used in 81 publications.

anti-CD45-PerCP-Cy5.5, clone 30-F11, BD Biosciences, Cat: 550994, Lot: 9249193
The manufacturer provides the following statement: Flow cytometry (Routinely Tested). The manufacturer states that the antibody was used in 151 publications.

anti-CD49b-PE, clone DX5, BioLegend, Cat: 108907, Lot: B278345
The manufacturer provides the following statement: Each lot of this antibody is quality control tested by immunofluorescent staining with flow cytometric analysis. For flow cytometric staining, the suggested use of this reagent is ≤ 0.25 µg per 106 cells in 100 µl volume. It is recommended that the reagent be titrated for optimal performance for each application. The manufacturer states that the antibody was used in 48 publications.

anti-F4/80-APC, clone BM8, BioLegend, Cat: 123116, Lot: B321485
The manufacturer provides the following statement: Each lot of this antibody is quality control tested by immunofluorescent staining with flow cytometric analysis. For flow cytometric staining, the suggested use of this reagent is ≤ 0.25 µg per 106 cells in 100 µl. It is recommended that the reagent be titrated for optimal performance for each application. The manufacturer states that the antibody was used in 402 publications.

anti-Gr-1.AF488, clone RB6-8C5, BioLegend, Cat: 108417, Lot: B297805
The manufacturer provides the following statement: Each lot of this antibody is quality control tested by immunofluorescent staining with flow cytometric analysis. For flow cytometric staining, the suggested use of this reagent is ≤ 0.25 µg per 106 cells in 100 µl volume. It is recommended that the reagent be titrated for optimal performance for each application. The manufacturer states that the antibody was used in 42 publications.

anti-CD3e, clone 145-2C11, BD Biosciences, Cat: 553057, Lot: 0121757
The manufacturer provides the following statement: Flow cytometry (Routinely Tested), Immunohistochemistry-frozen (Tested During Development), (Co)-stimulation, Blocking, Cytotoxicity, Fluorescence microscopy, Immunoprecipitation, Western blot (Reported). The manufacturer states that the antibody was used in 3 publications.

anti-CD4-PerCP-eFluor710, clone RM4-5, invitrogen, Cat: 46-0042-82, Lot: 4317308
The manufacturer provides the following statement: This RM4-5 antibody has been tested by flow cytometric analysis of mouse spleen cells. This can be used at less than or equal to 0.125 µg per test. A test is defined as the amount (µg) of antibody that will stain a cell sample in a final volume of 100 µL. The manufacturer states that the antibody was used in 116 publications.

anti-CD8a-Pacific blue, clone 53-6.7, BioLegend, Cat: 100725, Lot: B340544
The manufacturer provides the following statement: Each lot of this antibody is quality control tested by immunofluorescent staining with flow cytometric analysis. The manufacturer states that the antibody was used in 38 publications.

anti-CD16/CD32, clone 93, invitrogen, Cat: 14-0161-86, Lot: 2297433
The manufacturer provides the following statement: The 93 antibody has been tested by flow cytometric analysis of mouse splenocytes. This can be used at less than or equal to 0.5 µg per test. A test is defined as the amount (µg) of antibody that will stain a cell sample in a final volume of 100 µL. The 93 antibody has been reported for use in flow cytometric analysis, and has also been reported in blocking of Fc-mediated reactions in functional studies. For flow cytometric analysis, the manufacturer lists 394 publications.

anti-CD28, clone: 37.51, invitrogen, Cat: 14-0281-86, Lot: 2124568
he manufacturer provides the following statement: The 37.51 antibody has been tested by flow cytometric analysis of mouse splenocytes. This can be used at less than or equal to 0.5 µg per test. A test is defined as the amount (µg) of antibody that will stain a cell sample in a final volume of 100 µL. The 37.51 antibody has been reported for use in flow cytometric analysis, immunoprecipitation, and immunohistochemical staining. 37.51 has also been reported in costimulation of T cells in vitro and in vivo. For flow cytometric analysis, the manufacturer lists 80 publications.

anti-CD45-BV510, clone 30-F11, Biolegend, Cat: 103138, Lot: B386738
The manufacturer provides the following statement: Each lot of this antibody is quality control tested by immunofluorescent staining with flow cytometric analysis. The manufacturer states that the antibody was used in 130 publications.

anti-CD107a-FITC, clone eBio1D4B, BD Bioscience, Cat: 553793, Lot: 1356288
The manufacturer provides the following statement: Applications Tested: This antibody conjugate has been tested by intracellular immunofluorescent staining (≤ 1 µg/million cells, using the Cytofix/Cytoperm™ Kit, Cat. no. 554714) with flow cytometric analysis to assure specificity and reactivity. The manufacturer states that the antibody was used in 3 publications.

anti-IFNy-PE, clone XMG1.2, BioLegend, Cat: 505808, Lot: B352685
The manufacturer provides the following statement: Each lot of this antibody is quality control tested by intracellular immunofluorescent staining with flow cytometric analysis. For flow cytometric staining, the suggested use of this reagent is ≤0.25 µg per million cells in 100 µl volume. The manufacturer states that the antibody was used in 184 publications.

anti-IL-2-APC, clone JES6-5H4, BioLegend, Cat: 503810, Lot: B281632
The manufacturer provides the following statement: Each lot of this antibody is quality control tested by intracellular immunofluorescent staining with flow cytometric analysis. For flow cytometric staining, the suggested use of this reagent is ≤ 0.25 µg per 106 cells in 100 µl volume. The manufacturer states that the antibody was used in 22 publications.

anti-TNFα-PECy7, clone MPG-XT22, BioLegend, Cat: Cat: 506324, Lot: B338090
The manufacturer provides the following statement: Each lot of this antibody is quality control tested by intracellular immunofluorescent staining with flow cytometric analysis. For flow cytometric staining, the suggested use of this reagent is ≤0.25 µg per million cells in 100 µl volume. The manufacturer states that the antibody was used in 38 publications.

anti-CD4-AF488, clone GK1.5, BioLegend, Cat: 100423, Lot: B256618
The manufacturer provides the following statement: Each lot of this antibody is quality control tested by immunofluorescent staining with flow cytometric analysis. For flow cytometric staining, the suggested use of this reagent is ≤0.06 µg per million cells in 100 µl volume. The manufacturer states that the antibody was used in 33 publications.

anti-CD11a-eFluor450, clone M17/4, invitrogen, Cat: 48-0111-82, Lot: 1935030
The manufacturer provides the following statement: This M17/4 antibody has been tested by flow cytometric analysis of mouse splenocytes. This can be used at less than or equal to 0.125 µg per test. A test is defined as the amount (µg) of antibody that will stain a cell sample in a final volume of 100 µL. For flow cytometric analysis, the manufacturer lists 6 publications.

anti-CD44-APC, clone IM7, BioLegend, Cat: 103018, Lot: B317762
The manufacturer provides the following statement: Each lot of this antibody is quality control tested by immunofluorescent staining with flow cytometric analysis. For flow cytometric staining, the suggested use of this reagent is ≤ 0.25 µg per 106 cells in 100 µL volume. The manufacturer states that the antibody was used in 15 publications.

anti-CD45.2-PE/Dazzle594, clone 104, BioLegend, Cat: 109846, Lot: B319002
The manufacturer provides the following statement: Each lot of this antibody is quality control tested by immunofluorescent staining with flow cytometric analysis. For flow cytometric staining, the suggested use of this reagent is ≤0.25 µg per million cells in 100 µl volume. The manufacturer states that the antibody was used in 13 publications.

anti-CD69-PerCP/Cy5.5, clone H1.2F3, BioLegend, Cat: 104522, Lot: B287423
The manufacturer provides the following statement: Each lot of this antibody is quality control tested by immunofluorescent staining with flow cytometric analysis. For flow cytometric staining, the suggested use of this reagent is = 0.25 µg per 106 cells in 100 µl. The manufacturer states that the antibody was used in 28 publications.

anti-CD103-BV605, clone 2E7, BioLegend, Cat: 121433, Lot: B330662
The manufacturer provides the following statement:
Each lot of this antibody is quality control tested by immunofluorescent staining with flow cytometric analysis. For flow cytometric staining, the suggested use of this reagent is ≤0.5 µg per million cells in 100 µl volume. The manufacturer states that the antibody was used in 8 publications.

anti-CD127-FITC, clone A7R34, BioLegend, Cat: 135008, Lot: B255263

The manufacturer provides the following statement:
Each lot of this antibody is quality control tested by immunofluorescent staining with flow cytometric analysis. For flow cytometric staining, the suggested use of this reagent is ≤1.0 µg per million cells in 100 µl volume. The manufacturer states that the antibody was used in 15 publications.

anti-CXCR3-APC-Fire750, clone 173, BioLegend, Cat: 126539, Lot: B319572
The manufacturer provides the following statement:
Each lot of this antibody is quality control tested by immunofluorescent staining with flow cytometric analysis. For flow cytometric staining, the suggested use of this reagent is ≤0.25 µg per million cells in 100 µl volume. The manufacturer states that the antibody was used in 7 publications.

anti-IFITM3-Biotin, clone aa2-57, R&D Systems, Cat: BAF3377, Lot: XHC016041
The manufacturer provides the following statement: This antibody has been selected for use as a detection antibody in mouse IFITM3 Western blots and can be used at 0.1 - 0.2 µg/mL with the appropriate secondary reagents to detect mouse IFITM3.

anti-KLRG1-PE-Cy7, clone 2F1, invitrogen Cat: 25-5893-82, Lot: 1982690
The manufacturer provides the following statement: This 2F1 antibody has been tested by flow cytometric analysis of mouse splenocytes. This can be used at less than or equal to 0.25 µg per test. A test is defined as the amount (µg) of antibody that will stain a cell sample in a final volume of 100 µL. The manufacturer states that the antibody was used in 25 publications.

anti-P2X7R-PE, clone 1F11, BioLegend, Cat: 148704, Lot: B278527
The manufacturer provides the following statement: Each lot of this antibody is quality control tested by immunofluorescent staining with flow cytometric analysis. For flow cytometric staining, the suggested use of this reagent is ≤0.25 µg per million cells in 100 µl volume. The manufacturer states that the antibody was used in 1 publication.

Streptavidin-BV711, BioLegend, Cat: 405241, Lot: B370754
The manufacturer provides the following statement: Each lot of this Streptavidin-Brilliant Violet 711™ is quality control tested by immunofluorescent staining with flow cytometric analysis. The concentration provided is based upon molecular mass of streptavidin independent of any additional molecular mass that might be added by the Brilliant Violet 711™ conjugation. For flow cytometric staining, the suggested use of this reagent is ≤0.125 µg per million cells in 100 µl volume. The manufacturer states that the antibody was used in 14 publications.

anti-mouse IgA-FITC, Fortis Life Sciences, Cat: A90-103F, Lot: 21
The manufacturer provides the following statement: The accessible pdf document on the manufacturers webpage certifies that this product has met all of the quality control standards defined by Bethyl Laboratories, Inc. By immunoelectrophoresis and ELISA this antibody reacts specifically with mouse IgA. Cross reactivity to mouse IgM, IgG1, IgG2a, IgG2b, IgG2c, IgG3 and IgE is less than 1%. Some hybridoma clones may express aberrant immunoglobulin-related peptides that are improperly recognized by this antibody. The manufacturer states that the antibody was used in 3 publications.

anti-mouse IgG-FITC, clone Poly4060, BioLegend, Cat: 406001, Lot: B325073
The manufacturer provides the following statement: Each lot of this antibody is quality control tested by immunofluorescent staining with flow cytometric analysis. For flow cytometric staining, the suggested use of this reagent is ≤ 0.5 µg per 106 cells in 100 µl volume. The manufacturer states that the antibody was used in 14 publications.

anti-mouse IgG1-APC, clone RMG1-1, BioLegend, Cat: 406610, Lot: B302145
The manufacturer provides the following statement: Each lot of this antibody is quality control tested by immunofluorescent staining with flow cytometric analysis. For flow cytometric staining, the suggested use of this reagent is ≤0.25 µg per million cells in 100 µl volume. The manufacturer states that the antibody was used in 25 publications.

anti-mouse IgG2a-PerCP-eFluor710, clone m2a-15F8, invitrogen, Cat: 46-4210-82, Lot: 2464459
The manufacturer provides the following statement: This m2a-15F8 antibody has been tested by flow cytometric analysis of cells stained with a mouse IgG2a primary antibody. This can be used at less than or equal to 0.25 µg per test. A test is defined as the amount (µg) of antibody that will stain a cell sample in a final volume of 100 µL.

TotalSeqTM-C0301 anti-mouse Hashtag 1 Antibody, BioLegend, Cat: 155861, Lot: B362113
The manufacturer provides the following statement: Each lot of this antibody is quality control tested by immunofluorescent staining with flow cytometric analysis and the oligomer sequence is confirmed by sequencing. The manufacturer states that the antibody was used in 4 publications.

TotalSeqTM-C0302 anti-mouse Hashtag 2 Antibody, BioLegend, Cat: 155863, Lot: B375383
The manufacturer provides the following statement: Each lot of this antibody is quality control tested by immunofluorescent staining with flow cytometric analysis and the oligomer sequence is confirmed by sequencing. The manufacturer states that the antibody was used in 3 publications.

TotalSeqTM-C0303 anti-mouse Hashtag 3 Antibody, BioLegend, Cat: 155865, Lot: B368268
The manufacturer provides the following statement: Each lot of this antibody is quality control tested by immunofluorescent staining with flow cytometric analysis and the oligomer sequence is confirmed by sequencing. The manufacturer states that the antibody was used in 2 publications.

TotalSeqTM-C0304 anti-mouse Hashtag 4 Antibody, BioLegend, Cat: 155867, Lot: B361361
The manufacturer provides the following statement: Each lot of this antibody is quality control tested by immunofluorescent staining

with flow cytometric analysis and the oligomer sequence is confirmed by sequencing. The manufacturer states that the antibody was used in 1 publication.

anti-CD8a-BV421, clone 53-6.7, BioLegend, Cat: 100737, Lot: B284315
The manufacturer provides the following statement: Each lot of this antibody is quality control tested by immunofluorescent staining with flow cytometric analysis. For immunofluorescent staining using the µg size, the suggested use of this reagent is ≤0.5 µg per million cells in 100 µl volume. The manufacturer states that the antibody was used in 85 publications.

anti-CD8a-AF488, clone 53-6.7, BD Biosciences, Cat: 557668, Lot: 2070069
The manufacturer provides the following statement: Flow cytometry (Routinely Tested), Immunofluorescence (Tested During Development). The manufacturer states that the antibody was used in 25 publications.

anti-CD8a-AF674, clone 53-6.7, BioLegend, Cat: 100727, Lot: B375103
The manufacturer provides the following statement: Each lot of this antibody is quality control tested by immunofluorescent staining with flow cytometric analysis. For flow cytometric staining, the suggested use of this reagent is ≤ 0.25 µg per million cells in 100 µL volume. The manufacturer states that the antibody was used in 37 publications.

anti-CD45R/B220-AF488, clone RA3-6B2, BioLegend, Cat: 103225, Lot: B235299
The manufacturer provides the following statement: Each lot of this antibody is quality control tested by immunofluorescent staining with flow cytometric analysis. For flow cytometric staining, the suggested use of this reagent is ≤ 2.0 µg per million cells in 100 µl volume. For immunohistochemistry on frozen tissue sections, a concentration range of 2.5 - 5.0 µg/ml is suggested. For 3D immunohistochemistry on formalin-fixed tissues, a concentration of 5.0 µg/mL is suggested. The manufacturer states that the antibody was used in 44 publications.

anti-CD45R/B220-BV711, clone RA3-6B2, BioLegend, Cat: 103255, Lot: B326247
The manufacturer provides the following statement: Each lot of this antibody is quality control tested by immunofluorescent staining with flow cytometric analysis. For flow cytometric staining, the suggested use of this reagent is ≤0.25 µg per million cells in 100 µl volume. The manufacturer states that the antibody was used in 7 publications.

anti-APC-AF647, clone 936809, R&D Systems, Cat: FAB8927R, Lot: AFJR0120061
The manufacturer provides the following statement: Detects Allophycocyanin in flow cytometry. Detection of Allophycocyanin (APC) in Human PBMCs stained with APC-Cy7-Conjugated Anti-Human CD4 by Flow Cytometry. Human peripheral blood mononuclear cell (PBMCs) stained with APC-Cy7-conjugated Mouse Anti-Human CD4 Monoclonal Antibody were stained with (A) Mouse Anti-Allophycocyanin Alexa Fluor® 647-conjugated Monoclonal Antibody (Catalog # FAB8927R) or (B) isotype control antibody (IC0041R). Staining was performed using our Staining Membrane-associated Proteins protocol.

anti-mouseIgG2b-AF647, invitrogen, Cat: A21242, Lot: 2465093
The manufacturer provides the following statement: For stable signal generation in imaging and flow cytometry, Alexa Fluor 647 dye is pH-insensitive over a wide molar range. Probes with high fluorescence quantum yield and high photostability allow detection of low-abundance biological structures with great sensitivity. Alexa Fluor 647 dye molecules can be attached to proteins at high molar ratios without significant self-quenching, enabling brighter conjugates and more sensitive detection. The degree of labeling for each conjugate is typically 2-8 fluorophore molecules per IgG molecule; the exact degree of labeling is indicated on the certificate of analysis for each product lot. (tested: Western Blot, Immunohistochemistry, Immunocytochemistry)

Streptavidin-PE, Miltenyi, Cat: 130-106-790, Lot: 5230309005
The manufacturer provides the following statement: Human peripheral blood mononuclear cells (PBMCs) were stained with CD4-Biotin antibodies followed by labeling with Streptavidin conjugates. Flow cytometry was performed using the MACSQuant® Analyzer. The manufacturer states that the antibody was used in 2 publications.

All antibodies were titrated in house to optimize specific staining.

Eukaryotic cell lines

Policy information about cell lines

Cell line source(s)

- HEK293 cells (ATCC CRL-1573)
- HEK293A-HA (H1N1/PR/8) generated in house by stable transduction of HEK293 cells leading to HA H1N1/PR/8 expression
- HEK293A-HA (H3N2/HK/68) generated in house by stable transduction of HEK293 cells leading to HA H3N2/HK/68 expression
- HEK293A-NP generated in house by stable transduction of HEK293 cells leading to NP
- MDCK-II cells (ATCC CRL-2936)

Authentication

None of the cell lines were authenticated since their purchase.

Mycoplasma contamination

Cell lines were all tested negative for mycoplasma contamination.

Commonly misidentified lines
(See ICLAC register)

No commonly misidentified lines were used in this study.

Animals and other organisms

Policy information about studies involving animals; ARRIVE guidelines recommended for reporting animal research

Laboratory animals	Mus musculus, BALB/cJRj, female, 5-7 weeks, housed in individually ventilated cages under barrier, specific pathogen-free (SPF) S2 conditions in accordance with German law and institutional guidelines under specific pathogen-free (SPF) conditions with constant temperature (20°C-24°C) and humidity (45%-65%) on a 12h/12h-light/dark cycle
Wild animals	The study did not involve wild animals.
Field-collected samples	The study did not involve samples collected from the field.
Ethics oversight	The study was approved by the Government of Lower Franconia, which nominated an external ethics committee that authorized the experiments. Studies were performed under the project license AZ 55.2.2-2532-2-1081. The research staff was trained in animal care and handling in accordance to the FELASA and GV-SOLAS guidelines.

Note that full information on the approval of the study protocol must also be provided in the manuscript.

Flow Cytometry

Plots

Confirm that:

- [x] The axis labels state the marker and fluorochrome used (e.g. CD4-FITC).
- [x] The axis scales are clearly visible. Include numbers along axes only for bottom left plot of group (a 'group' is an analysis of identical markers).
- [x] All plots are contour plots with outliers or pseudocolor plots.
- [x] A numerical value for number of cells or percentage (with statistics) is provided.

Methodology

Sample preparation	Lungs were harvested and cut into small pieces followed by incubation for 45 min at 37 °C with 250 units Collagenase D and 80 units DNase I in 2 ml R10 medium (RPMI 1640 supplemented with 10 % FCS, 2 mM L-Glutamine, 10 mM HEPES, 50 µM β-mercaptoethanol and 1 % penicillin/streptomycin). Digested lung tissues were mashed through a 70 µm cell strainer before the single cell suspensions were subjected to an ammonium-chloridepotassium lysis. Approx. 15% of the total lung cell suspension were plated per well in a 96-well round-bottom plate for in vitro restimulation and phenotype assays.
Instrument	- ThermoFisher AttuneNxt 4 Laser (violet, blue, yellow, red) Acoustic Focusing Cytometer, Model AFC2, 2018, Serial 2AFC219120118, REF 4486521<br>- ThermoFisher AttuneNxt CytKick AutoSampler, Serial 2AAS400060323, REF A38975
Software	FlowJo v.10.8.1<br>FlowLogic v.7.2.1
Cell population abundance	CD45.2+, NP147-155-specific CD8+ T cells were sorted at a MoFlo Astrios Cell Sorter (Beckmann Coulter) in the core unit for cell sorting and immunomonitoring of the Friedrich-Alexander-Universität Erlangen-Nürnberg. For both groups of immunized or infected mice, 2x10^5 barcoded NP147-155-specific CD8+ T cells were sorted, the post-sort fraction was processed (10x Genomics library preparation and sequencing) and scRNA-seq and VDJ libraries were sequenced by Novogene UK. As the cells were intended for scRNA analysis, the generated count matrix was loaded into Scanpy. Doublets (two hashtags in one droplet) as well as negatives (no hash could be called) were removed with HashSolo (invoked via Scanpy), and cells with a mitochondrial content higher than 10% were filtered out. The defined target population still contained 91.01% of all barcodes initially present.
Gating strategy	Gating for intracellular cytokine staining after peptide-restimulation: Gating of singlets, lymphocytes, vital cells (dead lymphocytes were excluded by staining for the used live-dead marker), CD4/CD8 T cells (plotting CD8 against CD4), and iv-/iv + cells was similar as described below. Cytokine-producing cells were gated in parallel on the same hierarchy level for each analyzed parameter (CD107a, TNFa, IFNg, IL-2 for CD8; TNFa, IFNg, IL-2 for CD4), both in iv- and iv+ lung cells without consideration of the iv-staining. Control samples without stimulation, anti-CD3-stimulated samples, and controls without cytokine staining were used to set the gate for the respective cytokine-positive population. In order to quantify polyfunctional cells positive for all assessed markers, as well as functional subpopulations, the implemented Boolean gating function of FlowJo was used.<br><br>Gating of antigen-experienced memory CD8+ T-cell subsets: Doublets were excluded by plotting FSC-H against FSC-A followed by gating on cells with roughly similar height and area. Next, only cells highly positive for CD45.2 were selected, while dim and negative cells were not included. Lymphocytes were gated by plotting FSC-A against SSC-A. Small FSC debris was excluded as well as high SSC and high FSC events. Gating was checked by backgating of T lymphocytes. Definition of

stained/unstained cells was done with the help of unstained controls. Next, CD8 T cells were gated by plotting FSC-A against CD8 signal. The population was discrete and well defined. Unstained controls did not show any signal. Subsequently, pentamer positive cells were defined. Only cells highly positive for the specific MHC-II pentamer were selected, while dim and negative cells were not included. Pent+ CD8+ T cells were then divided into iv+ and iv- cells based on staining for the in vivo injected anti-CD45 antibody. Both populations were well separated from each other. TRM phenotypes were determined within the iv- population and the circulating phenotypes (TEFF, TEM, TCM) were defined within the iv+ population. TEFF and TEM were identified by their expression of KLRG1 and presence (TEM) or absence (TEFF) of CD127. KLRG1-negative cells were then gated and CD69 was plotted against CD103. CD69-CD103- cells were gated and TCM within the iv+ population were defined by their expression of CD127. CD127+ TCM were defined at an identical cut-off as for the TEM cells and this cut-off was determined in comparison to unstained controls. TRM were defined as CD69+/-CD103+/- cells within the iv- KLRG1- compartment.

Gating of antigen-experienced memory CD4+ T-cell subsets: Gating of singlets, CD45.2-positive cells, lymphocytes, CD4+ T cells was similar as described below. Definition of stained/unstained cells was done with the help of unstained controls. The populations were discrete and well defined. Unstained controls did not show any signal. Next, CD44+ cells were defined. Only cells highly positive for CD44 were selected, while dim and negative cells were not included. CD44+CD4+ T cells were then divided into iv+ and iv- cells. based on staining for the in vivo injected anti-CD45 antibody. Both populations were well separated from each other. CD4 TRM phenotypes were determined within the iv- population by their expression of CD69, CD103 and/or CD11a.

☒ Tick this box to confirm that a figure exemplifying the gating strategy is provided in the Supplementary Information.
